# Migraine prevalence, alexithymia, and post-traumatic stress disorder among medical students in Turkey

**DOI:** 10.1007/s10194-012-0452-7

**Published:** 2012-04-26

**Authors:** Hatice Balaban, Murat Semiz, İlteriş Ahmet Şentürk, Önder Kavakçı, Ziynet Çınar, Ayfer Dikici, Suat Topaktaş

**Affiliations:** 1 Department of Neurology, Faculty of Medicine, Cumhuriyet University, 58140 Sivas, Turkey; 2 Department of Psychiatry, Faculty of Medicine, Cumhuriyet University, 58140 Sivas, Turkey; 3 Department of Biostatistics, Faculty of Medicine, Cumhuriyet University, 58140 Sivas, Turkey

**Keywords:** Prevalence, Migraine, Post-traumatic stress disorder, Alexithymia, Comorbidity, Medical students

## Abstract

The aim of this study was to investigate the prevalence of migraine, alexithymia, and post-traumatic stress disorder among medical students at Cumhuriyet University of Sivas in Turkey. A total of 250 medical students participated in this study and answered the questionnaires. The study was conducted in three stages: the self-questionnaire, the neurological evaluation, and the psychiatric evaluation. In the first stage, the subjects completed a questionnaire to assess migraine symptoms and completed the three-item Identification of Migraine Questionnaire, the Toronto Alexithymia Scale, and the Post-Traumatic Stress Disorder Checklist-Civilian Version Scale. The subjects who reported having a migraine underwent a detailed neurological evaluation conducted by a neurologist to confirm the diagnosis. In the final stage, the subjects with a migraine completed a psychiatric examination using the structured clinical interview for DSM-IV-R Axis I. The actual prevalence of migraine among these medical students was 12.6 %. The students with a migraine were diagnosed with alexithymia and post-traumatic stress disorder more frequently than those without migraine. The Migraine Disability Assessment Scale scores correlated with the post-traumatic stress disorder scores. The results of this study indicate that migraine was highly prevalent among medical students in Turkey and was associated with the alexithymic personality trait and comorbid psychiatric disorders including post-traumatic stress disorder. Treatment strategies must be developed to manage these comorbidities.

## Introduction

Migraine and tension-type headache are the most common primary headache types. Migraine is a neurologic disorder that represents a significant health problem due to the frequency and accompanying morbidity that includes disability and loss of performance [1–3]. Migraine has a lifetime prevalence of 12–18 %, which has been shown to be both age and gender dependent in community-based studies worldwide [4]. The lifetime prevalence of migraine in Turkey was found to be 10.9 % in men and 21.8 % in women in a nationwide epidemiological study [5]. In studies, a higher percentage of migraine headache have been reported in women; female hormones may be a factor responsible for the sex difference.

Previous studies have demonstrated an association between migraine and specific psychiatric disorders in the general population. Although the association between migraine and depression is the most widely reported, there are also strong associations between migraine and other psychiatric disorders and conditions [6–10]. Researchers have reported that anxiety disorder, post-traumatic stress disorder (PTSD), depression, and alexithymia are more frequently reported in adults with migraine [11–16].

Although the association among various psychiatric comorbidities and migraine is well established in adult case–control studies, the prevalence of these disorders in university students with migraine has not been examined in detail.

The social distribution of migraine has long been a subject of speculation. Epidemiological studies help clinicians understand the frequency and nature of the pain. Studies have also shown such interesting aspects as the risk factors related to socio-demographic status and genetic and environmental factors in populations [1, 17]. The epidemiology of migraine among medical students is of particular interest because migraine is the most common type of headache in young adults. Psychological and physical stressors are more common in medical students than the general population [18]. These stressors could be triggers for migraine in this population. The migraine prevalence was reported to be between 12.4 and 21.9 % in Turkey using self-reporting measures [1, 2, 19]. Prevalence studies reported that migraine was highly prevalent among university students and was associated with impaired academic performance [1, 2, 19–21]. The results of studies in various countries have consistently shown that migraine sufferers report limited daily activities at work or at school [21]. In university students, a decreased school performance limits success, which may influence their future occupational performance. Furthermore, both migraines and the majority of psychiatric disorders are reported to begin during the college years. A detailed investigation of associated psychiatric conditions in university students is warranted because psychiatric comorbidities are already associated with poor performance in the activities of daily living among migraine sufferers [21].

PTSD is a condition in which an overwhelming traumatic event results in intense fear, helplessness, horror, and avoidance of stimuli associated with the trauma [13]. PTSD has been shown to worsen the chronicity and disability of patients with chronic pain. Pain and pain-related disability could be reduced with treatment for PTSD [16, 22]. de Leeuw et al. [14] reported that the consistency of PTSD symptoms appears to be more frequent in patients with recurrent headache than in the healthy population. Afari et al. [13] also found that PTSD symptoms following combat injury were associated with headache. A higher frequency of PTSD was reported in patients with migraine headache than the general population [16]. Peterlin et al. [15] reported an increased prevalence for PTSD in episodic migraine patients compared with subjects without headache in their study which is the largest and most substantial study on PTSD and migraine in the literature.

Interestingly, some symptoms that resemble PTSD including nightmares, intrusive visual images, insomnia, depression, and learning impairments have been frequently reported among medical students [23]. However, a correlation between the PTSD symptoms and migraine is still an understudied topic in medical students.

Alexithymia is a personality trait that is conceptualized as deficits in a person’s ability to employ cognitive processes to identify, differentiate, and communicate one’s affective states [24, 25]. Alexithymia and PTSD have been frequently reported among patients with chronic pain [24]. Interestingly, previous studies have found a correlation between alexithymia and PTSD symptoms. Studies have reported an association between a perceived difficulty in identifying and describing emotional states and the severity of PTSD symptoms [26, 27].

Although migraine is also prevalent among medical faculty, health sciences, and psychology students, previous studies have not adequately assessed comorbid psychiatric conditions and personality traits in this specific population [28, 29]. Researchers have reported the prevalence of migraines using self-reporting instruments without performing neurological evaluations. Therefore, reliable studies in which individuals diagnosed with migraine are subjected to a detailed evaluation by a neurologist and psychiatrist are needed. Understanding the nature of the association between migraines and psychiatric disorders and conditions has implications for diagnosis and treatment. The occurrence of comorbidity may also provide clues to the aetiology of each disorder. The aim of this study is to investigate the actual prevalence of migraine and the comorbidity of alexithymia and PTSD among medical students at Cumhuriyet University of Sivas in Turkey.

## Materials and methods

### Participants and study design

The study was conducted with students of the Medical Faculty at Cumhuriyet University in Sivas, Turkey during the 2010–2011 school years. There are a total of 850 students at the Medical Faculty. Of these 850 students, 250 were selected with randomized stratified sampling (*p* = 0.15, α = 0.01, and *d* = 0.05). Of the 250 students, 11 refused to participate in the study. To reach the 250 participant count, we gave the questionnaires to the next students when a student did not want to participate. The study was approved by the Medical Faculty Hospital Ethics Committee and consent was obtained from each subject.

The study incorporated three stages. In the first stage, the students were asked to complete a standardized questionnaire to establish a migraine diagnosis during a school visit (Appendix). The first part of the instrument consisted of questions regarding demographic characteristics including age, medical history, family history, family structure, family socioeconomic status, smoking habits, and alcohol use. The second part was composed of questions related to the 2004 diagnostic criteria for the International Headache Society (IHS) for migraines [30]. The ID Migraine, Toronto Alexithymia Scale (TAS), and PTSD Checklist-Civilian Version (PCL-C) were also used.

The subjects who reported migraine in the screening questionnaire participated in the second stage of the study. During this stage, a neurologist conducted a full neurological evaluation to confirm the migraine diagnosis. The neurological evaluation detailed a headache history and a neurological examination. The Turkish version of the Migraine Disability Assessment Scale (MIDAS) questionnaire was then administered to the students to assess failure due to migraine. In the third stage, students with migraine completed a psychiatric examination with the Structured Clinical Interview for DSM-IV-R Axis I (SCID-I). The psychiatric examination was performed by two psychiatrists (MS, AD). In addition, 31 students without migraine but with similar age and gender were selected randomly to complete the SCID-I. Finally, the SCID-I results of the groups were compared.

### Materials

#### Identification of migraine (ID Migraine™)

As a widely used screening instrument for identifying migraine at primary health services, the ID Migraine is a three-question screening tool for migraines that has demonstrated good validity [31]. Each of the three items relates to a central diagnostic symptom of migraine: nausea, photophobia, and interference with activities. Each question is scored dichotomously with endorsements of two or more items suggesting probable migraine sensitivity and specificity. A positive predictive value on this test has been defined as 81, 75, and 93 %, respectively. The Turkish version of the ID Migraine™ screening test has previously been validated [32].

#### Toronto Alexithymia Scale (TAS-20)

The prevalence of alexithymia was investigated using the 20-item version of the Toronto Alexithymia Scale (TAS-20), Turkish version [33–35]. The three dimensions of the TAS-20 are the following: (1) difficulty identifying feelings, (2) difficulty describing feelings, and (3) externally oriented thinking. The total scores for the TAS-20 were categorized with a score ≥61 indicating alexithymia and a score of <61 indicating no alexithymia [35].

#### PTSD Checklist-Civilian Version (PCL-C)

The PCL-C is a self-report measure used to assess the incidence of significant stressors and the prevalence of PTSD symptoms [36]. The Turkish version of the PCL-C was performed by Kocabaşoğlu et al. [37]. Prior to completing this questionnaire, the respondent is asked to identify significant traumatic stressors from a 15-item list that includes experiences such as military combat, violent attack, incarceration, natural or man-made disaster, severe auto accident, sudden injury/serious accident, observing someone else being hurt or killed, and learning that your child has a life-threatening illness [14].

#### Migraine Disability Assessment Scale (MIDAS)

The MIDAS questionnaire is used to gather information on a disability in terms of missed days of paid work (or school), housework (chores), and non-work time. The questions are asked regarding either days of missed activity or days during which productivity was reduced by at least 50 %. If productivity decreased to 50 % or less, the day is considered missed [38]. The 4-point grading system for the MIDAS questionnaire is as follows: Grade I (scores ranging from 0 to 5), little or no disability; Grade II (scores ranging from 6 to 10), mild disability; Grade III (scores ranging from 11 to 20), moderate disability; and Grade IV (scores of 21 or greater), severe disability. The Turkish version of the MIDAS questionnaire was developed by Ertaş et al. [39].

#### The Visual Analogue Scale (VAS)

The VAS is a simple and commonly used method for evaluating variations in pain intensity [40]. The subjects are instructed to indicate the intensity of their pain by marking a 100-mm line anchored with terms that describe the extremes of pain intensity.

#### The structured clinical interview for DSM-IV-R (SCID) I

According to the DSM-IV, the SCID-I is a clinical interview comprised six structured modules that are utilized by an interviewer to determine whether an individual has one or more Axis-I disorders. The average application period is 25–60 min, and the evaluation is conducted with the patient individually. During the application, the interviewer uses an administration booklet with interview questions and a scoring sheet to record ratings. The psychiatric diagnosis is determined based on “current” and “lifetime” experiences [41]. Developed by First et al. in 1997 into a Turkish reliability study, an adaptation of the SCID-I was conducted by Özkürkçügil et al. [42]. For all diagnoses, the interviewer agreement was 98.1 %, and the kappa coefficient was 0.86. For all diagnostic categories, the kappa coefficients changed between 0.52 and 1.00 and were significant (*p* < 0.001).

### Statistical analyses

Statistical analyses were performed using the Statistical Package for the Social Sciences (SPSS) Version 14.00. The data of categorical variables were presented as counts and percentages; the data of continuous variables were presented as the mean and SD. A comparison of variables between the groups was performed using the Independent *t* test for numeric variables and the χ^2^ test for categorical data. First, the correlations between the MIDAS scores and the PTSD scores and the MIDAS scores and the alexithymia scores were tested using linear regression analysis. Then, a multivariate step-wise linear regression analysis was applied to the MIDAS scores, the PTSD scores and the alexithymia scores in students with migraines. In all analyses, *p* values below 0.05 were considered significant.

## Results

A total of 250 students answered the questionnaires in this study. Four 17-year-old students were excluded from the analysis. In total, 246 students between the ages of 18 and 26 years with a mean age of 20.85 ± 2.10 years were eligible in this study. Of the 246 students, 127 (52 %) were female, and 119 (48 %) were male. Table 1 displays the demographic and social characteristics of the 246 students.

**Table 1:** Demographic characteristics of students

	Total	Migraine	Non-migraine
Number	246	31	215
Age	20.85 ± 2.10	20.90 ± 1.70	20.84 ± 2.10
Sex			
Female	127 (51.6 %)	25 (80.6 %)	102 (47.4 %)
Male	119 (48.4 %)	6 (19.4 %)	113 (52.6 %)
Marital status			
Single	241 (97.9 %)	30 (97 %)	211 (98.1 %)
Married	5 (2.1 %)	1 (3 %)	4 (1.9 %)
Family history of headache			
Yes	85 (34.6 %)	22 (71 %)	63 (29 %)
No	161 (65.4 %)	9 (29 %)	152 (71 %)
Family history of neurological disease			
Yes	4 (1.7 %)	0 (0 %)	4 (1.9 %)
No	242 (98.3 %)	31 (100 %)	211 (98.1 %)
Smoking			
Yes	22 (9 %)	4 (13 %)	18 (8.4 %)
No	224 (91 %)	27 (87 %)	197 (91.6 %)
Alcohol use			
Yes	22 (9 %)	6 (19.3 %)	16 (7.4 %)
No	224 (91 %)	25 (80.7 %)	199 (92.6 %)

Migraine-type headache were detected in 34 of 246 subjects using a self-reporting instrument; however, only 31 of the 34 subjects were diagnosed with migraine based on a personal neurological interview. Therefore, 215 (87.3 %) of the 246 subjects were migraine-free. The self-reported migraine prevalence was 13.8 %, whereas the actual migraine prevalence among the medical students was 12.6 %. Of these 31 subjects with migraine, 25 (80.6 %) were female, and 6 (19.4 %) were male. The mean age of disease onset was 16.87 years, and the average number of attacks per month was 5.96 (min 1–max 15). The mean pain intensity was 5.48 (min 3–max 9). In addition, 27 (87.1 %) of 31 students had migraine without aura, whereas the remaining 4 students (12.9 %) had migraine with aura. No differences were found between the socioeconomic status of those students with migraine and those without. Similarly, smoking habits and alcohol use did not differ significantly between the groups. Table 2 shows the clinical characteristics of the migraine.

**Table 2:** Clinical characteristics of migraine in students

Characteristics	Number/Total
Pain level	
Mild	10/31
Moderate	16/31
Severe	5/31
Frequency of pain at 3 months	
0–5	16/31
6–10	14/31
11	1/31
Mean duration of attacks	
4–5 h	13/31
6–11 h	3/31
12–23 h	4/31
24 h or more	11/31
Types of migrane	
With aura	4 (12.9 %)
Without aura	27 (87.1 %)

Only 3 of the students with migraine had previously consulted a physician for their headaches, whereas the remaining 28 students had not. None of the 31 students with migraine were being treated for the condition. Similarly, none had visited a psychiatrist prior to the study.

The MIDAS scores showed that 13 (41.9 %) students had minimal disability with a mean score of 3. Five (16.1 %) students had mild disability with a mean score of 8. Six (19.3 %) had moderate disability with a mean score of 13, and finally, seven (22.5 %) had severe disability with a mean score of 25 (Table 3).

**Table 3:** MIDAS grades of the students

MIDAS grade	Number *n* (%)
Grade I	13 (42)
Grade II	5 (16)
Grade III	6 (19)
Grade IV	7 (23)

PTSD was found in 13 (5.2 %) of the 246 subjects and was not detected in 233 of the 246 subjects. Of the 31 students with migraines, 7 (22.5 %) were found to have PTSD, whereas 6 (2.8 %) of the 215 migraine-free students had PTSD (odds ratio = 10.16, 95 % CI = 3.16–32.71, *p* = 0.001). A significant correlation was found between MIDAS scores and PTSD scores by linear regression analysis (*t* = 2.95, *r* ^2^ = 0.23, *r* = 0.48 *p* = 0.006).

Alexithymia was found in only 9 (3.6 %) of the 246 subjects. However, alexithymia was detected in 4 (12.9 %) of the 31 students with migraine. Only 5 (2.3 %) of the 215 migraine-free students had alexithymia (ODS = 6.33, 95 % CI = 1.57–24.60, *p* = 0.01). The students with migraine had significantly higher rates of alexithymia (Table 4). The results of linear regression analysis showed a correlation between alexithymia and MIDAS sores (*t* = 2.40, *r* ^2^ = 0.16, *r* = 0.41 *p* = 0.023).

**Table 4:** Association between migraine, PTSD, and alexithymia

	With migraine	%	Without migraine	%	Total	*p*
PTSD-by PCL-C						
Yes	**7**	22.5	6	2.8	13	<0.001
No	24	77.5	209	97.2	233	
PTSD-by SCID-I						
Yes	4	12.9	0	0	4	<0.001
No	27	87.1	31	100	58	
Alexithymia						
Yes	4	12.9	5	2.3	9	<0.01
No	27	87.1	210	97.7	237	

*PTSD-by PCL-C* diagnosis of post-traumatic stress disorder by *PTSD* Checklist-Civilian Version, *PTSD-by SCID-I* diagnosis of post-traumatic stress disorder by Structured Clinical Interview for DSM-III-R Axis I

Finally, a multivariate linear regression model was designed between alexithymia scores, PTSD scores, and MIDAS scores. PTSD scores correlated with MIDAS scores (*t* = 2.06, *r* ^2^ = 0.27, *r* = 0.55, *p* = 0.04); a multivariate regression analysis did not show a correlation between alexithymia and MIDAS scores.

When the results obtained using the SCID-I were examined, a current SCID-I psychiatric diagnosis was found in 9 (29 %) of the 31 subjects with migraine and 2 (6.45 %) of the migraine-free subjects (χ^2^ = 5.41, *p* = 0.02). A total of 15 (48.4 %) of the 31 students with migraine were found to have had a lifetime SCID-I psychiatric diagnosis, whereas only 6 (19.4 %) of the students without migraine had a lifetime SCID-I psychiatric diagnosis. When the two groups were compared in terms of lifetime SCID-I diagnosis, the difference was significant (χ^2^ = 5.83, *p* = 0.01) (Table 5).

**Table 5:** Results of the SCID-I evaluation of the students

	SCID-I			
	Current SCID-I		Lifetime SCID-I	
	Migraineurs	Nonmigraineurs	Migraineurs	Nonmigraineurs
PTSD	4		4	
Panic dis	2	1	2	1
Depression	1	1	4	3
Spec phobia	1		1	1
Soc phobia			1	
Dysthymia	1			
Gen anx dis			2	1
OCD			1	
Total	9	2	15	6

*PTSD* post-traumatic stress disorder, *panic dis* panic disorder, *spec phobia* specific phobia, *soc phobia* social phobia, *gen anx dis* generalized anxiety disorder, *OCD* obsessive compulsive disorder

## Discussion

The present study examined migraine prevalence and its association with PTSD and alexithymia in a sample of 246 medical students. PTSD and alexithymia were found to be significantly higher in a selected cohort of medical students. The subjects with migraines were interviewed and evaluated by a neurologist and a psychiatrist in this study. The diagnosis of migraine was determined using the 2004 IHS criteria, which are utilized in epidemiological studies due to their high sensitivity [30]. The prevalence of migraine was found to be 12.6 %. Migraine without aura was the most common type of migraine found in this study. The observed migraine prevalence was similar to those reported in larger population studies that used structured diagnostic interviews. The prevalence of migraine has been reported to be between 12.4 and 21.9 % among university students in Turkey [1, 2, 19]. However, the prevalence of migraine among medical students reportedly ranges from 11 to 40 % worldwide [3, 28, 29]. A higher prevalence has been reported in some studies. The differences may be attributed to the methodological differences among these studies. Different self-reporting questionnaires may result in different prevalence values. The use of a self-administered questionnaire might lead to misunderstanding of some questions with the risk of certain subjectivity in the answers. The examination of subjects by a neurologist enabled the confirmation of the screening results and the exclusion of other types of headache.

PTSD occurs as a result of exposure to extreme traumatic stressors that arouse feelings of intense fear, helplessness, and horror in exposed individuals [22]. There is increasing support in the literature for a strong association between migraine and PTSD in adult migraine patients. High PTSD prevalence rates of 22–50 % have been reported in individuals with migraine in previous studies of adult subjects fulfilling the criteria for PTSD [13, 14, 22].

In an examination of the diagnostic overlap between PTSD and migraines, Peterlin et al. [16] reported that migraine sufferers with PTSD had a significantly greater disability than those without PTSD. However, Ifergane and colleagues [43] also examined the relationship between PTSD and migraine, and they reported that the prevalence of PTSD in migraine patients was not significantly different than in the general population. The prevalence of PTSD with migraine was studied in a large general population sample of over 5,600 adults. The authors found a 12-month prevalence of PTSD to be 14.3 % in episodic migraine patients compared to 2.1 % in those with no headaches. In addition, the life-time prevalence of PTSD was 21.5 % in episodic migraineurs compared to 4.5 % in subjects without headaches. An increased prevalence of PTSD in chronic daily headache patients was also reported in that study [15]. The results of the present study showed that PTSD with migraine was as high in medical students as in the general population.

The exact pathophysiological mechanism of migraine and associated PTSD is not precisely known. An increased sensitivity to stressors has been described in migraine and may be related to PTSD pathophysiology. Alterations in serum serotonin levels have been linked to both PTSD and migraine pathogenesis. The most widely accepted theory is dysfunction of the hypothalamic- pituitary- adrenal axis [15, 44–47].

People with alexithymia have difficulty in expressing their feelings. The term was initially used to denote an adaptive style that creates a tendency to develop psychosomatic symptoms [11, 48]. Several studies have analysed the prevalence and impact of alexithymia among chronic pain patients, and these studies have resulted in a better understanding of the relationship between chronic pain and personality traits [24, 25]. A limited number of studies have examined the correlation between alexithymia and migraine.

Wise et al. [49] studied anxiety, depression, and alexithymia in patients with tension-type headache and migraine. The authors found no difference between subjects diagnosed with migraine and those with tension-type headache with regards to alexithymia, levels of anxiety or depression. In contrast, Lumley et al. [50] reported that the alexithymia personality trait has a negative impact on the pain severity of chronic pain patients including migraine headaches. Muftuoglu et al. [11] found that migraine patients were considerably more depressed, anxious, and alexithymic than the healthy controls. Yalug et al. [12] reported a significantly higher score in measures of depression but not alexithymia or anxiety in chronic migraine patients compared to episodic migraine patients. However, significant correlations were noted between depression, anxiety, and alexithymia in the study by Yalug et al. A high correlation has been reported among alexithymia, anxiety, and depression scores, which may indicate that alexithymia is associated with psychological distress. Our findings also indicate that alexithymia is common among medical students with migraine.

In the present study, the prevalence of PTSD and alexithymia was significantly higher in subjects with migraine than in those without migraines. The results of screening tests revealed that seven students with migraines had PTSD. Nevertheless, when those students were evaluated using the SCID-I, only four of them met the criteria for a PTSD diagnosis. This difference highlights the fact that self-report tests and psychiatric interviews yield different prevalence rates as stated in the “Introduction” of this paper. The data obtained from self-reporting evaluations may help to detect cases under the threshold but may also be limited to the perceptions of the individual, making them unreliable.

The results of studies conducted in different countries support the association between migraine, PTSD and alexithymia in adults. In the present study, higher rates of PTSD and alexithymia were found in students with migraines than in migraine-free students. Interestingly, a high rate of alexithymia has been reported in PTSD cases [26, 27, 48]. Previous studies have shown a relationship between PTSD and alexithymia. This relationship needs to be explained in migraineurs.

The results of several studies have shown that PTSD has a negative impact on the abilities of chronic pain patients. In this study, a significant correlation was found between the MIDAS disability score and the PTSD score in students with migraine. The students with migraine appear to be characterized by a higher prevalence of PTSD and alexithymia than individuals in the general population.

One strength of the present study is that the students diagnosed with migraine were evaluated by a neurologist and a psychiatrist in the second and third stages of the study, respectively, which allowed for the diagnoses of migraine headache and psychiatric conditions to be performed based on objective measures. On the other hand, this study has some limitations. First, only 31 migraineurs students were detected in this selected population, and this count may not allow our results to be generalizable and may not represent the whole population. Moreover, we evaluated alexithymia in students only by a self-reported questionnaire, and we did not investigate the association between alexithymia scores and PTSD scores.

In conclusion, both PTSD and alexithymia are common in medical students with migraine, and PTSD symptoms correlate with pain-related disability. The finding that the majority of the students with migraine had not visited a neurologist or a psychiatrist shows that there is a lack of diagnosis and treatment in this population. The identification and treatment of PTSD and alexithymia in medical students with migraine are an important and potentially modifiable health state. Taken together, the results of previous studies with adults and the present study suggest that the treatment of PTSD and alexithymia positively influence the levels of pain and migraine-related disability in medical students.

## Appendix

See Table 6.

**Table 6:** Migraine headache questionnaire form

Subject number:				
Name:				
Class:				
Age:				
Gender:	Female	Male		
Marital status:	Single	Married		
Family type:	Nuclear	Extended	Other	
Socioeconomic status:	Lower	Middle	Upper	
Headache history in family:	Yes	No		
History of neurological disease in family:	Yes	No		
Your systemic disease:	Yes	No		
Your psychiatric disease:	Yes	No		
Smoking habits:	Yes	No		
Alcohol use:	Yes	No		
Have you ever had five or more headache unrelated to any other illness during the last 6 months?				
	Yes	No		
Headache characteristics:	Throbbing	Burning	Pressing	
Localization:	Unilateral	Bilateral		
Mean duration of headache:				
Associate symptoms:	Nausea	Vomiting	Photophobia	Phonophobia
Pain intensity:	Mild	Moderate	Severe	
Aggravation by or causing avoidance of physical activity:	Yes	No		
